# Food, fields and forage: A socio-ecological account of cultural transitions among the Gaddis of Himachal Pradesh in India

**DOI:** 10.1016/j.heliyon.2021.e07569

**Published:** 2021-07-12

**Authors:** Aayushi Malhotra, Sailaja Nandigama, Kumar Sankar Bhattacharya

**Affiliations:** Department of Humanities and Social Sciences, Birla Institute of Technology and Sciences Pilani, Pilani Campus, Rajasthan, India

**Keywords:** Traditional food systems, Socio-ecology, Cultural change, Land-use pattern, Gaddi Community

## Abstract

Traditional food systems of many ethnic communities in India directly depend on their symbiotic relationship with the surrounding natural resources and the local socio-ecological and cultural dynamics. However, in the light of development activities resulting in drastic socio-ecological changes, these communities are oftentimes found stranded with over-simplified and unsustainable food systems. Using an ethnographic methodology, we present the case of Gaddis – an agro-pastoral community of Himachal Pradesh in India. In this paper, we documented the on-going trade-offs in traditional livelihoods of the Gaddis and their land use patterns that cause a significant transition in the traditional food systems. Based on our observations, we argue that mapping the shifting political ecology of resources enables a better understanding of transitioning food systems and the consequent eco-cultural changes. While doing so, we emphasize the need for revisitng the existing praxis of tribal development in India with an urgent focus on holistic socio-ecological approaches.

## 1 Introduction

India is a melting pot of cultures with a diversity of ethnic communities residing across its geography. Some of these communities, depending on criteria like socio-economic backwardness, geographic isolation, and cultural distinctiveness, are designated as Scheduled Tribes under Article 342 of the Indian constitution. Majority of these communities reside in vulnerable socio-ecological zones and practice traditional occupations that are in-sync with their natural environment. These occupations enable them to have symbiotic relationships with their local ecology and play a vital role in shaping their food systems. These traditional food systems (hereafter TFSs) are oftentimes the outcomes of time-tested and culturally accepted resource dependence patterns. They form the socio-ecological interface highlighting the relationships that the tribal communities have with their local ecologies. They are also reflective of the diverse provisioning skills, techniques, and cultural beliefs of the people (Kwik, 2008). Simultaneously, TFSs play a role in strengthening the cultural identities embedded in local worldviews and maintaining social cohesion at the community level (Kuhnlein et al., 2013; Ohna et al., 2012).

However, TFSs of the tribal communities are now found to be dwindling in the light of the current development scenario that favours the forces of industrialisation and urbanisation that result in cultural and environmental homogenisation. Numerous alterations in the distinct domains of traditional livelihoods, land use patterns and resource availability are collectively affecting the TFSs. It was also observed that the local tribes are rather quickly assimilating the mainstream food cultures due to their easy availability through Public Distribution System (PDS) since last few decades. Such practices are not only against the health of the coupled human-environment arrangements but also put the community-based resource management in jeopardy. Our study maps such declining TFSs of the Gaddi tribe of Himachal Pradesh (HP) in India, to comprehend the larger implications of such phenomena on the Gaddi socio-ecological and cultural systems.

Gaddi^1^ is a traditional agro-pastoral community located in the western belt of middle Himalayas. The centre of residency and fabled ancestry for all the Gaddi population spread across the state of Himachal Pradesh (hereafter HP) remains in the Bharmour region in Chamba District. This region enjoys a status of the scheduled area under the Indian Constitution, where the Gaddi population remains numerically and politically a dominant Scheduled Tribe (Thakur and Sharma, 2012). Owing to this, the region, and the community, both receive a greater development focus from the state government under the Integrated Tribal Development Project (ITDP) and Tribal Sub-Plan (TSP)^2^. These development measures undertaken in the past few decades have led to major socio-economic shifts in the region further intensifying the process of cultural change among the Gaddis. This paper also probes deep into this process of change in terms of the altering TFSs through a close analysis of the catalysts of change.

Although a significant body of literature exists on the Gaddis (Bhasin, 2013: John and Badoni 2013; Pandey, 2015; Thakur and Kahlon 2015; Saberwal, 1996), the transition among their traditional food systems is rather thinly documented. This sparse literature captures the significance of the Gaddi subsistence agriculture and pastoral practices from both the cultural as well as the ecological viewpoints but overlooks the relation these practices have with their TFS. Gaddi TFS has remained rooted in their transhumant agro-pastoral practices for generations. The dual enterprise of livestock rearing, and subsistence cultivation not only provided them with food security but also ensured the required monetary gains in an ecologically sustainable manner. It helped them cope with the restrictions that the difficult terrain and extreme weather conditions put on their agricultural pursuits. Consequently, the Gaddi developed a seasonal pattern of natural resource utilisation that offers them several socio-cultural by-products and also ensures profitable returns in slack seasons. However, this strategy of diversification despite its acknowledged socio-ecological importance has come under tremendous pressure in the face of the mainstream economic choices provided by the new development arrangements.

Himachal Pradesh is engaged in a rapid expansion of road network for the past few decades, largely driven by the setting up of several hydro-electric power projects, mineral and slate mines along with the booming apple cultivation. These developments, on the one hand, promoted economic diversification in the state and on the other hand led to a decline in traditional occupations, and food production practices (John and Badoni 2013; Thakur and Kahlon 2015). While the growing regional infrastructure and development of socio-economic conditions of people are seen by the state as positive outcomes, the inevitable negative impacts that these developments brought along are always under-represented. In this paper, we highlight the significant decline in the TFS of the Gaddis, which is one such negative effect for the local socio-ecology and the local culture. We trace this trend to be rooted in the political ecology of the above mentioned development regime in the region.

Along with mapping the pattern of Gaddi livelihood transformations that puts their traditional food systems in peril, we also consider the government interventions that look after the infrastructure of food production and distribution. For instance, measures such as the Green Revolution^3^ and Public Distribution System^4^ (PDS) exemplify the steps taken in this direction by the government of India (Bhasin, 2013). These measures primarily aimed at fulfilling the needs of an exponentially growing population, in process, ignored the linkages local food systems have with their specific socio-cultural and ecological environments. They also obscure the underlying fact that food is much more than a commodity for the traditional communities like the Gaddi. Over a period of time, this kind of obscuration contributed to the declining TFS and downgraded the sustainable food production practices making the community more vulnerable and less self-sustainable. This resulted in an unprecedented and increased dependency of the Gaddis on the market and the government subsidies, thereby, considerably weakening their traditional self-sufficient and resource-efficient food systems. Hence, even if the structural changes seem to provide short-term economic gains to the Gaddi community, these gains nevertheless remain highly unsustainable in the broader socio-ecological and cultural context.

In this paper, we highlight the transitions in TFS of the Gaddis to articulate their changing socio-ecological landscape. According to the scientific studies (e.g., Kuhnlein and Receveur 1996; Temple and Steyn 2016), TFS are capable of providing better nutrition, enabling the sustainable development and preventing the emergence of lifestyle diseases that have their origin in the phenomenon of modern economic growth. Nevertheless, pure advocacy for the promotion of traditional diets remains out of the scope for this paper. Instead, we define food systems in this study as not only limited to the choices of edible articles, but also covering the processes around food production, distribution, and procurement. The purpose of this study is to situate the TFS in the larger socio-ecological practices and arrangements followed by the Gaddis that are the result of their historical understanding of the environment and their subsistence needs. Therefore, by considering food system as an essential signifier of socio-cultural and ethnic identities (Kuhnlein and Receveur, 1996; Ohna et al., 2012), the study outlays the on-going cultural change among the Gaddis in India.

This paper is developed into five sections. The first section covers an elaborate introduction that conceptually and geographically contextualises the study, followed by the second section, which presents a thematic literature review. The third section of this paper discusses the methodological orientation and procedures of data collection. Findings of the research are thematically arranged in the penultimate section while the analysis and articulation of the concluding observations are accommodated in the fifth section.

## 2 Conceptual and thematic literature review

### 2.1 Food as a significant part of the culture

Food patterns specific to any community develop through several generations of lifestyle practices. Many contextual socio-ecological factors influence the way these practices evolve over a period of time. Literature enlists local practices based on ethnic identities, agro-climatic conditions, socio-cultural ethos and religion as essential factors that play a role in shaping the food choices of people (Savitri and Bhalla 2007). Apart from these, the economic and political reasons are also responsible for the food-related decisions one makes. Kwik (2008) calls such choices and practices as Traditional Food Knowledge (hereafter TFK). Under this knowledge system, local communities develop a particular cultural acceptance towards food systems that relate to the local natural resources and their seasonal availability (CLAXTON, 1998; Kuhnlein and Receveur, 1996). These systems remain economically and ecologically sensible and vulnerable to the extent of getting affected by external development measures.

According to Samuel and Makhani (2016), food does not merely provide nutrition and life sustenance but also holds intrinsic cultural value for the concerned communities. Therefore, it should be understood as part of a cultural fabric that forms the traditional heritage and knowledge repository for any community (Kwik, 2008; Settee, 2011; Watts, 2008). As a whole, TFK systems entail comprehensive socio-cultural meanings that consequently co-shape the socio-ecology of any place. The study of food patterns concerning the culture helps in "highlighting the changing material conditions, scientific and medical understandings as well as political and social contingencies of complex societies" (Watts, 2008, p. 368).

### 2.2 Transitioning food and cultural patterns

According to Phillips (2006), to comprehend the cultural change interlinked with the food systems, one must focus on how the food is produced, processed, distributed and purchased. A constellation of factors is responsible for shifting cultures both locally and globally. The multiplicity of influences including demographic changes, increasing demands, global governance, environment and social movements play an essential role in deciding the ways in which food systems advance. The integration of the world's cultures through globalisation has 'deterritorialised' the food patterns (Phillips, 2006), creating a disconnect with the local TFK. Simultaneously, technological improvements and changes in agricultural practices also drive similar types of transitions. Although these changes are integral for meeting the increasing demands of rising populations, the resultant modern food systems nevertheless remain unsustainable and problematic (Kearney, 2010; Schutter, 2017) primarily due to their disconnect with the socio-cultural and ecological roots of the communities.

There are extensive records of how the traditional multi-crop regimes viably provide varied and nutritious food along with the much-needed by-products that can be recycled and reused for sustainable ends (Edison and Devi, 2019; Kattides and Lima, 2008; Welthungerhilfe, 2016). In the recent past, the decline in the TFSs has become a norm rather than an exception. The changing production processes are also held responsible for declining agrobiodiversity in various regions (Galipeau, 2015; Schutter, 2017). Crop monocultures often pushed under the rubric of the green revolution, and genetically modified organisms dominate modern food production practices. These monoculture practices often result in a skewed valuation of cropland residues as waste material instead of a valuable and re-useable resource (CLAXTON, 1998). It not only adds up to the environmental burden but also results in a simultaneous drop in the cultivation of ecologically suitable and sustainable variety of native crops (Schutter, 2017).

A significant factor that contributes to the declining TFS is the state-run food security intervention through public distribution systems (PDS). While introducing diets of the dominant cultures among indigenous societies (Kuhnlein and Receveur, 1996) these programmes often do not take the local food practices and people's preferences at the grassroots into account. Finnis (2007) calls such transitions to be rooted in the discourse of political ecology that manifests through the existing development programmes and not alone through market integration. According to her research, documenting the social memory of food helps in understanding the micro perspective of political ecology of dietary and agricultural transitions. Additionally, Settee (2011) in her study on indigenous knowledge, mentions that the traditional food that is very much a part of the indigenous wisdom is fast disappearing as a consequence of land degradation that usually accompanies the industrial and other types of development projects. Multiple factors responsible in fading traditional food systems include-global industrialisation, monocultures, food advertising, urbanisation, cultural homogenisation, shifting diets and consolidation of food chains (Galipeau, 2015; Kattides and Lima, 2008; Kuhnlein and Receveur, 1996; Kwik, 2008). With an overwhelming presence of these factors, many indigenous communities that have remained directly dependent on their habitus and natural environment for their food and livelihoods are getting adversely impacted. The drastic modifications in the food systems are also resulting in major nutritional transitions with side effects on the health of the local people as well as on their immediate environments (Edison and Devi, 2019; Kearney, 2010; Loring and Gerlach, 2009; Schutter, 2017). Such shifts are known to produce food system imbalances while simultaneously affecting the health of the communities in the long run (Loring and Gerlach, 2009). Often these changes culminate in loss of the symbiotic relationship between people and their natural environments (Kuhnlein and Receveur, 1996), which remains integral for a sustainable socio-ecological balance, and prevention of drastic climate imbalances. Therefore, developing a holistic understanding of the cultural changes vis-à-vis the changing TFS becomes essential to mitigate the loss of socio-ecological balance.

## 3 Methodology

This study is an outcome of an on-going ethnographic doctoral project on the Gaddis of Himachal Pradesh in India. The qualitative data presented in this paper was collected throughout 2019 in a phased manner from the villages of Bharmour tehsil^5^ in Chamba district of Himachal Pradesh. After building up rapport with the respondents in the initial phases of the fieldwork, data was collected through recurring household visits and meetings organised at public places including community grounds and temples within the villages.

Following a combination of convenient and snowball sampling method, qualitative data collection tools including in-depth interviews (*n = 20)*, oral histories (n = 5) and focus group discussions (FGDs) (n = 3) were periodically conducted. All the willing members of the Gaddi community, irrespective of their intersectional differences, were approached for their consent to participate in the study. Consent was obtained verbally or by getting the informed consent forms signed by the participants. No specific exclusion criteria was devised for the respondents, as their physical availability oftentimes remained challenging in the difficult terrain of the study site following their seasonal mobility and the shifting settlements of their residence^6^. The data used in formulating the arguments of this paper is extracted from a larger pool of information drawn for studying the socio-ecological transitions in the region. Changing food practices and the cultural implications emerged as an offshoot of the thematic analysis of the qualitative information collected using the ethnographic immersion techniques such as participant observation and in-depth interviews with the Gaddis, and the oral histories with their elders.

To highlight the diachronic changes in the food systems, oral history accounts of the community elders were taken into consideration while analysing the periodical and generational changes in the food culture of the Gaddis. According to Zerubavel (1996), the social memory of the elders offers a reliable baseline to determine these socio-cultural changes over their lifetime. Therefore, their lived experiences become vital for documenting the specific cultural changes resulting in shifting food cultures and socio-ecological variations. Additionally, participant observation and the techniques of ethnographic immersion were also used to provide a critical vantage point to the whole discussion.

A thorough review of secondary literature also helped in augmenting a diachronic understanding of the on-going cultural change as well as in substantiating the qualitative findings from the fieldwork. The available literature on the Gaddis sporadically captures the essential transitions taking place in the region but fails to put it in a holistic perspective of cultural change in general and TFS in particular. Through this study, we aim to address this gap by articulating the underlying cultural shifts in terms of changing TFS of the Gaddis. To comprehend such changes within the Gaddi community, we are drawing an inspiration from a conceptual framework constituted by Kuhnlein and Receveur (1996) in their comparative study on the indigenous food patterns and loss of use of traditional food systems. Their study collates the factors responsible for loss of indigenous food systems and projects the implications for several indigenous communities. It further helps us in initiating the arguments and assembling a nexus of factors resulting in wide-ranging cultural and socio-ecological changes among the Gaddis using traditional food patterns as a lens. The underlining assumptions of Kuhnlein and Receveur's conceptual framework guide our analysis in this paper. These include-firstly that the food-related needs are perceived to be multifactorial and dependent on the environmental, societal, and personal aspects. Secondly, the food systems of any given community are considered to be moderated by the current technology, politics, climate change, and development measures. Together these two assumptions help us in connecting the micro-level transformations with the systemic level variables, thus giving us a vantage point to map and analyse the changing food culture of the Gaddis over a period of time. Thematic analysis of the data using manual coding procedures provided us with the major themes that we discuss in the later sections of this paper.

Kuhnlein and Receveur (1996) gave a general account of the indigenous food system for the communities that live in conformity with their own social, cultural and economic traditions. This conceptual model not only helped in initiating our analysis but also extended our understanding of the food systems among the Indian tribal communities, that are broadly termed as Scheduled Tribes in India. These groups are classified with special constitutional status and welfare provisions. *Gaddi* of Himachal Pradesh are one such scheduled tribe with high numerical preponderance in the state. Their scheduled tribe status, along with their population strength and their geographical location, served as a significant selection criterion for this study. Additionally, the recently amplified development focus shown by the HP state government towards this region was a significant motivation to look into the on-going transitions.

## 4 Understanding Gaddi food system in transition

With the establishment of Integrated Tribal Development Project (ITDP) in Himachal Pradesh under the Tribal Sub Plan scheme of 1974, Gaddis started receiving an increased development focus from the state government (Negi, 1998; Thakur and Sharma, 2012). A nucleus budget scheme and a single line administration that streamlined the funds for welfare related activities were introduced in the tribal regions for faster dissemination of development policies (Negi, 1998). These measures introduced the tribal areas to external development agencies and increased the pace of change. With variable economic growth, tribal areas also got exposed to unintended socio-ecological consequences including the on-going transitions in their traditional food systems. We discuss such changes among the Gaddis in detail below-

### 4.1 Shifting traditional occupation and food production

Gaddi traditional agro-pastoral occupation forms an excellent example of a self-subsistent food system, as this pattern is primarily based on the seasonal use of resources and complementarity between agricultural and pastoral practices. It not only ensures food security but also works as an essential adaptive strategy that helps in efficient utilisation of irregularly distributed and least productive resources (Bhasin, 2013).

Traditionally, Gaddis rear sheep and goats by following a vertical seasonal transhumance pattern^7^ (Bhasin, 2013). The inadequate availability of grazing grounds along with the extreme winters in the Himalayan region of their residence is responsible for this mobility pattern. They are also known to practise subsistence agriculture in the semi-temperate zone of western Himalayas during the favourable season in their settled villages (Thakur and Kahlon 2015). The limited agrarian accomplishments of the Gaddi are oftentimes attributed to the disinterest of the population in carrying out the laborious farming practices limited by the agricultural capacity of their lands. Consequently, the production of native and resilient food crops along with meat and dairy production (goat milk) obtained through traditional agro-pastoral occupations remained central to their TFS. This provided them with the food and nutrition along with the organic fertilisers for their agriculture fields, and forage for their livestock. Therefore, it would not be wrong to say that the whole praxis of agro-pastoralism contributed in maintaining the mountainous socio-ecology and in co-shaping the TFS of the Gaddis.

Gaddi pastoral occupation includes a seasonal downhill migration, which enables their regular and periodical access to the local markets. This exchange also strengthens their reciprocal relationships with the sedentary agriculturalist populations of the plains (Bhasin, 2013; Pandey, 2015; Saberwal, 1996). Traditionally, this system of exchange helped Gaddis in procuring those food crops that were not ecologically supported by the higher altitude agricultural fields. The Gaddi in-turn provided the agriculturalists with the manure, wool and meat and also, helped them in their everyday domestic chores. Through these seasonal interactions, Gaddi pastoralists not only procured the agricultural residue as forage for their livestock but also were successful in collecting the food grains for their self-consumption. These practices indirectly supplemented the Gaddi traditional occupation as well as their TFS for ages.

However, it has been observed that in the last few decades, Gaddis are constantly abandoning their traditional subsistent agro-pastoralism to adopt commercial horticulture and other diversified livelihood practices. Unlike earlier, a significant portion of the land in the settled villages of Bharmour region is now under apple cultivation, leaving no fallow land or crop residues for livestock grazing (Thakur and Kahlon 2015). This directly affects the continuity and growth of their traditional agro-pastoral livelihoods. One of the Gaddi respondents expressed his concern regarding the proliferating fruit cultivation in the region as-

*"Currently, it's all apple cultivations everywhere in the region. Although the apple crop suits our land (which does not support extensive agriculture otherwise), it deprives us of our staple and traditional food articles. It may provide us with a pocket full of money, but at critical times of need, it would not give us proper food to eat. Bringing all the land under horticulture has left us with no scope for growing our traditional food these days. If in case some extreme natural calamity takes place and government supplies do not reach our region, we would be left with nothing to eat and all we will have remaining will just be the apples and the money"*. (Source: In-Depth interview with Karam Chand, an elderly male from the Gaddi community in Bharmour, June 2019)

The sentiments expressed above reflect the shifting cropping patterns and large-scale economic diversification resulting in changing land use patterns in the region at an accelerated pace. Maximum of the middle-aged Gaddi population we interacted with is now either engaged in private businesses or salaried jobs with less or no interest in cultivating their own fields. Furthermore, the young and the educated Gaddi are also seen to dissociate themselves with the agriculture fields and the traditional pastoral profession (John and Badoni, 2013). They would rather move out of the region in search of new opportunities in urban areas, than engage in their traditional occupations. Most of the elderly Gaddi respondents categorise these urban jobs as low paid and temporary while reinstating the importance of owning one's own flock and cultivating one's own food.

*"These jobs in the cities do not pay well and there remains a dangling sword on your head that you would be thrown out today or tomorrow. In our traditional agro-pastoralism, we are our own bosses. We can sell one animal if a need arises and manage any crisis with the income. What do these youngsters have! Even if they are earning in the cities, they ask for money from home."* (Source- In-depth interview with Sahab Singh, ex-pastoralist from Village Chobhia, Bharmour, June 2019. He favours agro-pastoral practices against the salaried jobs in the cities).

Outmigration of the Gaddi youth for city jobs, local economic diversification and shifts towards remunerative horticulture are some of the significant changes enlisted by our respondents as factors that affect their traditional occupation in the Bharmour region of Himachal Pradesh. These changes that now people are well attuned with have an otherwise more profound and invisible negative impact affecting the food systems of the Gaddi community. A substantial decline in agro-pastoral practices has thus influenced their traditional food production and procurement processes at various levels that we discuss in the next sections.

### 4.2 Changes in land-use practices

Himalayan ecology and terrain are critical factors that shape the TFS of Gaddis. The extreme seasonal variance and fragmented terrace cultivations do not allow for technological experimentation, which otherwise is possible in the plains. Therefore, for an extended period, Gaddi agricultural practices remained limited to production for household consumption only (Bhasin, 2013; Malhotra, 1935; Mukherjee, 1994). However, with the decline in traditional occupations, Gaddis are now inclined towards capitalising on their fragmented land assets. Almost every Gaddi household in Bharmour region possesses plots of land that are used for residence and agriculture purposes in varying proportions (Pandey, 2015). In several cases, the plots remain fragmented and agricultural fields lie distant from the houses. The inheritance pattern followed by the Gaddi allows a single person to own numerous small-scale land holdings across the village (Hänninen, 2014). Such a varied distribution of land also makes it unfavourable for carrying out large-scale traditional agricultural activities. With the shifting land-use strategies, acreage under agriculture is declining while the horticultural (mainly apple cultivation) activities are on a constant rise. This shift towards remunerative cultivation that diminishes the scope of growing traditional crops has a direct effect on the TFS of Gaddis.

Table 1 below shows the transitions in Gaddi land use pattern mainly highlighting the changes in horticulture practices in Bharmour region over the last four decades. The growing popularity of horticulture is also symbolic of shifting lifestyle priorities as the Gaddi households are now occasionally dependent on their land for staple food production. Paradoxically, one could simultaneously observe their increasing dependence on external sources for food procurement discussed in the next section.

**Table 1:** Changes in land use pattern at Bharmour, Himachal Pradesh, India.

Categories	Area in hectares (during 1978–79) (Mukherjee, 1994)	Area in Hectares (during 2016–17) (Government of Himachal Pradesh, 2017)
Net area sown	4106	4319
Horticulture (Fruit crops)	10	4769

Despite the limited seasonal agriculture, the staple food patterns of Gaddis traditionally included a diversity of food crops grown variably over Rabi and Kharif season in their local fields (Fieldwork, 2019). It mainly comprised of *makki* (Maize- Zea Mays), *gehu* (Wheat-Triticum aestivum), Barley (Hordeum vulgare), *Bhrase* (Buckwheat or Fagopyrum esculentum), *Chinae* (Hill millet or Panicum miliaceum), *Phullan* (millets), *Sieul* (Amaranthus amaranthoides), *Kodra* (Paspalum scrobiculatum) *Urad* (Phaseolus radiates) and *Kulth* (Dolichos biflorus) (Verma 1996; Thakur and Kahlon 2015). Along with these native food crops, local fruits also formed an essential part of Gaddi food system. Seeds from the seasonal fruits, including apricots *(cheedh)* and walnuts (*akhrot)* were used to obtain the cooking oil through traditional methods (Fieldwork Data, 2019). Additionally, the local staple diet also included good portions of meat, as it was readily available. However, with the declining pastoral profession, meat consumption has reduced because of the limited local supply and a drastic increase in its price.

Along with that, the changing land use pattern with more land coming under horticulture has also directly affected the local agricultural practices (Thakur and Kahlon 2015). Apple cultivation has come to occupy the prime focus of the Gaddis as a source of better income with lesser manual labour required in the annual cycle than what was required for traditional agricultural practices. However, this trend has also contributed to the declining scope for mixed farming as no secondary crop could sustain under the mature apple trees. As a result of this changing land-use pattern, the agricultural lands are continually coming under mono-cropping while the majority of the Gaddis stop cultivating the native food crops. Only a handful of households were growing the native crops for self-consumption at the time of this study in 2019. A similar situation was documented by another case study among the Hill Kolli Malayalis of Tamil Nadu, where the cash crops have replaced the cultivation of staple food articles like millets (Finnis, 2007). In case of Gaddis, similar trends are observed as they shift to more remunerative crops including apple, *Rajmah* (Phaseolus Vulgaris), Potatoes and few other varieties of lentils (Thakur and Kahlon 2015). In essence, these agrarian transitions are resulting in the disappearance of local and traditional food grains from the fields and plates of the Gaddis.

Under the changed agricultural practices and cropping patterns, organic manure is replaced by the increased use of chemical fertilisers, composts and pesticides for a better yield. These industry-produced synthetic substances are mainly acting as the substitutes for the essential by-products that were initially obtained from the livestock in the Gaddi villages. The traditional pastoral activities besides ensuring food security in the region, also supplemented the agriculture fields with rich organic manure. However, at present, with a tremendous decline in pastoral activities and increasing apple cultivation, which does not permit livestock grazing, the use of organic manure in the fields has significantly decreased. Moreover, these changes are facilitated by the government programmes that provide the chemical fertilizers for farm use on subsidized prices. Declining availability of organic manure and easy access to the chemical fertilizers have become the only resort for practicing Gaddi farmers to sustain their agricultural and horticultural produce. They are pushed to adopt such practices despite having a cursory knowledge about their detrimental outcomes to escape the topographic vulnerabilities as well as to maintain their household economy (Field observations, 2019).

Additionally, these changes in land use pattern are also affecting the social structure of the Gaddi community that remains closely associated with their TFS. Historically, the Gaddi community followed a joint family pattern where the land possessions were held within the extended family (Pandey, 2015). This pattern ensured ample availability of agricultural labour required to cultivate the jointly held land holdings while maintaining the community cohesion. The harvest was also shared within the family or even within the community.

However, with the changing times and increasing social distance within the families, the land has been further subdivided into fragmented holdings. This has led to an infrastructural change in the region where new houses and buildings are coming up to accommodate separate nuclear families that earlier lived collectively under one roof as one household. The disintegration of family structure has also created inadequacy of labour as well as land among the Gaddis, making it challenging for them to pursue their traditional subsistence agricultural activities.

### 4.3 State interventions and public distribution system

Amidst the socio-ecological and cultural changes stated above that adversely impact the local food production, another major area of concern remains the current source of food procurement. The response to this conundrum appears straightforward, however it remains multifaceted and layered in nature. Currently, the Gaddi community is relying on external sources for their food procurement in the absence of traditional agro-pastoral activities. These sources, including the expanding markets, and government run food security programmes like PDS have become the primary source and for some the only source for food procurement. Although PDS has worked a great deal in ensuring a continuous food supply, it ignores the socio-ecological significance of TFS of the Gaddi community. For instance, unlike their TFS, the PDS system operating in the Bharmour region supplies the residents with rice, lentils, wheat, sugar and cooking oil, that did not form a significant part of their staple food patterns till a few years back. All the food articles that the subsidised ration shops redistribute under PDS were alien to their TFS. Edison and Devi (2019) and Finnis (2007) also observed comparable transitions among the Adivasis of Attappady in Kerela and the Hill Kolli Malayalis of Tamil Nadu in India. These studies reinforced our findings of how the development policies of the government around food security, including the PDS, result in the drastic transition in TFSs.

The local Gaddi respondents also reported several repercussions in terms of health, land use and agricultural practices because of such drastic changes. One of the old male Gaddi respondents informed us that-

*"Everything we procure from the market is a product of chemicals. Even vegetables are injected with god knows what chemicals. It has led to many health-related issues in the people, including increased cases of sugar (Diabetes), high blood pressure and youth having no stamina. They (younger generation) can't even walk in the plains for a longer time let alone climb the mountains."* (Source- Nanak Chand, a local Gaddi resident of Bharmour in a FGD on changing agricultural activities in the region, July 2019).

**Figure 1:**
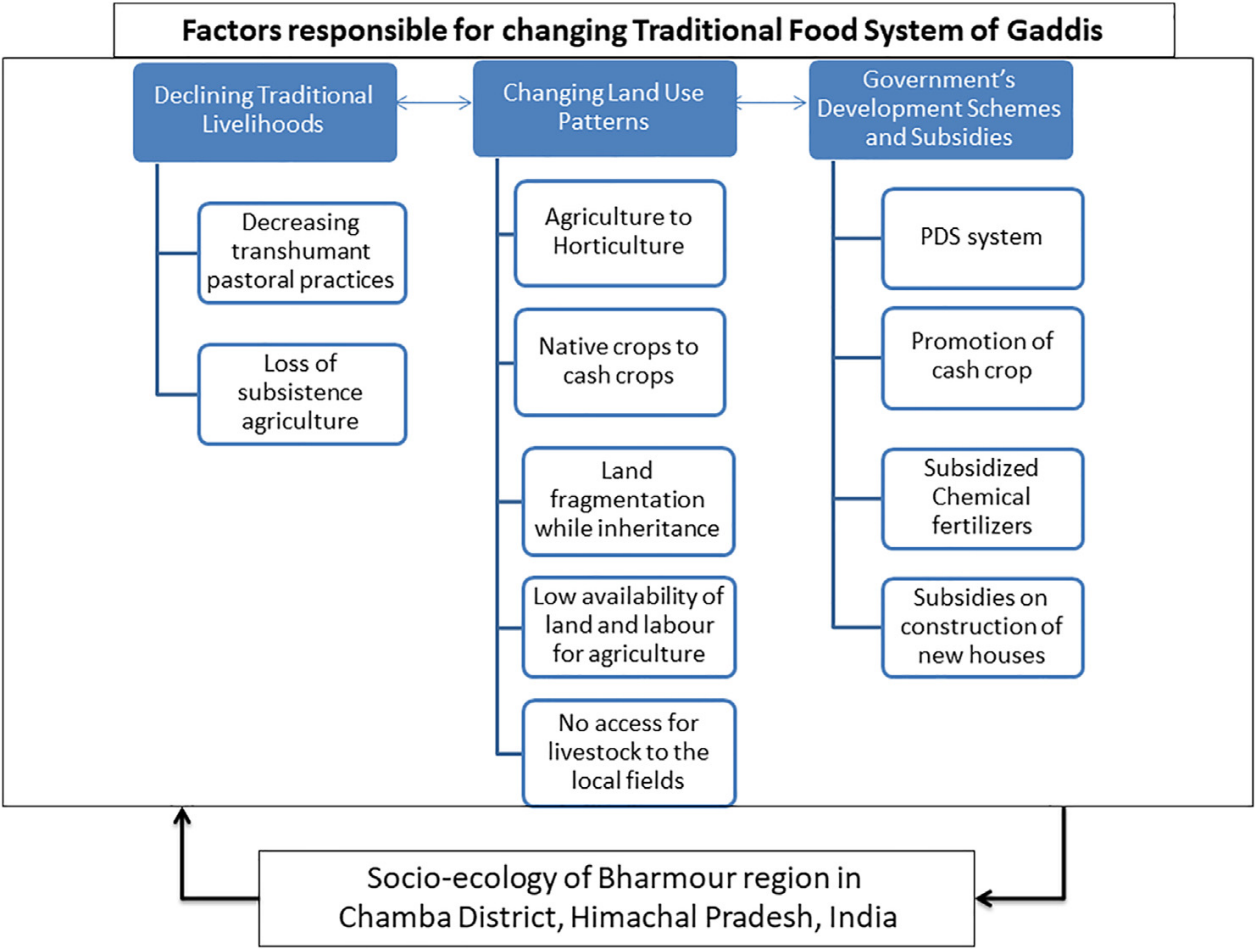
The changing socio-ecological and cultural landscape of Gaddis (field data collected in 2018–19).

To sum up our findings, the following table presents the changes that the Gaddi TFS has undergone (see Table 2). These changes correspond to the conceptual framework given by Kuhnlein and Receveur (1996). Collectively, it suggests that transitions in Gaddi TFS are directly associated with the modifications in discrete domains of their everyday lives. It includes livelihood strategies, land use patterns, family structures, social networks, as well as community and youth aspirations. All these inter-dependent domains get impacted by the on-going development activities in the region and contribute to socio-ecological and cultural change among the Gaddis. Figure 1 graphically illustrates these linkages in detail.

**Table 2:** The changing food system of the Gaddi community.

Variable processes in the Food systems	Traditional practices	Changes observed
Food Production	-Subsistence Agriculture (*Makki, Phoolan, Bhares, Chinae, Sieul, Jau*)	-Remunerative agriculture and Horticulture (*Rajmah, Makki, Aaloo,* Apple cultivation)
	-Meat and milk from livestock rearing	-Declining livestock rearing and increasing meat prices
Food Distribution or procurement	-Self-sufficient	-Increased dependency on government-run ration shops
	-Reciprocal exchanges within the community or with peasant community on migratory routes	- Increased purchases from the market
Food Consumption	Staple diets consisting of natively grown food crops and meat	-Processed and packaged food articles
		-Food grains mainly rice, wheat and lentils procured from ration shops

## 5 Concluding observations

It is evident that the Gaddis are rapidly transitioning to the diets of their surrounding dominant cultures without realising the long-term impacts of these changes on the quality of their life as well as their local socio-ecology. The traditionally grown food crops that included many climatically resilient varieties of millets are fast disappearing from their local food regimes. The same millets are now paradoxically emerging as 'superfoods' in the dominant food cultures of urban India. Gaddi community has started losing out on its traditional food culture and its agro-biodiversity only recently- but the steep transition curve that has come to stay in its place is indicating a trend observed throughout the global south. This trend points towards the irreversible loss of biological and ecological complexity at the regional level that adversely impacts the symbiotic relationship of communities (like the Gaddi) with their native habitats. This level of steep transition not only creates an imbalance, but also alienates the indigenous communities like Gaddis from their traditional habitats and practices.

Gaddi traditional agro-pastoral systems stressed the sustainable need fulfilment aspects (Bhasin 2013). However, the ongoing transitions in their livelihood strategies and land use patterns have disrupted this tradition. The declining pastoral profession also led to the demise of their age-old reciprocal relationship with the settled peasant populations. These relationships were not only fundamental in maintaining food security during pastoral migrations for the Gaddis, but also helped in creating a sustainable resource sharing mechanism across these communities and ecological zones. The seasonal crop residue from agricultural fields helped to feed the pastoral livestock during winters when the natural resource availability for them was otherwise limited. With the gradual decline of this reciprocal relationship, and the increased competition for resources, deterioration of the social networks and marginalisation of the traditional pastoral profession has come to occupy the void.

The political ecology of resource access and control determines the food choices that people make, and it also directs the course of cultural change within their communities (Finnis, 2007). The shifts as mentioned above in case of Gaddi TFS confirm this idea and highlight how such transitions are altering the traditional relationships between tribal communities and their local socio-ecological systems. The changes that initially appeared to be socio-economic have ultimately resulted in modifying the whole socio-ecological and cultural schema for the Gaddis. As a result, their TFS has deviated from being self-sufficient to that of being subservient to the external agencies. This unsustainable dependence for food production, distribution and procurement steered by the market and the government has come to characterize the steep curve of transition in the Gaddi TFS. The planned development interventions exogenously introduced in the region are upsetting the interconnected domains of everyday lives including land use pattern, livelihoods, and the social structure of the Gaddis. This resulted into a shifting cultural milieu for the Gaddi community (see Figure 1).

We do recognise that the socio-ecological practices change over a period of time for any given community (Nandigama, 2020). However, it becomes crucial to raise the alarm when these changes end up being unsustainable for the community's self-sufficiency, existence and continuity. Declining traditional food systems of the Gaddi raise a similar concern, as they are reflective of the diminishing socio-ecological balance and cultural stability. Also, this decline accelerates the process of cultural erosion (Handa, 2005) and leads to homogenisation and loss of diversity. The model of planned development that is continuing in case of the Gaddis not only dismantles their age-old practices of self –sufficiency and sustainability but also increases their external dependencies and vulnerabilities. In addition, the transitions recorded by us in the Gaddi food system also enhance the already existing fragility of the Himalayan ecosystems. Our analysis of these transitions, which otherwise ignore the adverse impact of the mainstream development activities among the tribal communities and their habitats, takes the debate on the nature of development to a next level where the whole idea of tribal development, as is adopted by the contemporary governments gets to be revisited. In the case of Gaddis, we noticed that the mainstream planned development processes inadvertently sabotaged their cultural sustenance and eco-cultural landscape, including the traditional food systems. The mainstream development processes ignored the local, community-based age-old practices that had the potential to reproduce sustainable resource regeneration and reuse practices in the face of meagre and limited ecological resources. This discussion leads to a pertinent query on whether the way forward for the world is to learn from the fast-disappearing sustainable lifestyles of the communities like the Gaddi or to continue jostling the planned yet inadvertently unsustainable development regimes. These unsustainable regimes further marginalize the already vulnerable communities suffering from the ecological and climate-based uncertainties at various scales. Through this paper, we provide a snapshot of how various socio-ecological domains and processes remain interconnected despite their perceived disconnect by the policy makers. This paper demonstrated how the dynamic and symbiotic functioning of a socio-ecological system spanned out over a period of time, using a lens of the traditional food systems of the Gaddis. We feel that a solution to such a problem should arise organically from within the Gaddi community, with the optimal support of the local governments and the other key policy makers who could proactively re-visit and re-route the ongoing tribal development programmes towards more sustainable and indigenous food cultures and technologies. Essentially, a holistic socio-ecological approach to development remains a viable step in this direction.

## Declarations

### Author contribution statement

Aayushi Malhotra: Conceptualization, Data collection, analysis and interpretation, Writing and revision.

Sailaja Nandigama: Conceptualization, Data analysis and interpretation, Writing and revision.

Kumar Sankar Bhattacharya: Data interpretation, Writing and revision.

### Funding statement

This research did not receive any specific grant from funding agencies in the public, commercial, or not-for-profit sectors.

### Data availability statement

Data will be made available on request.

### Declaration of interests statement

The authors declare no conflict of interest.

### Additional information

No additional information is available for this paper.

## Footnotes

1 The term '*Gaddi*' is used for community reference, while the people from the community are called as '*Gaddis*'.

2 Tribal Sub Plan were formulated under the fifth five-year plan for economic development of tribal population groups by the Government of India majorly focusing on the tribal economy and welfare. Areas with large concentration of tribal populations saw the establishment of ITDP's for better outreach of development incentives.

3 Green Revolution was initiated in 1960's in India for agricultural reformation by introducing the changes in ways of production like technological interventions, hybrid high yielding seeds etc.

4 PDS is a food security programme run by the government of India to redistribute the food grains at a fair price.

5 Tehsil is a smaller administrative division within a district. Bharmour Tehsil in Chamba district is a constitutionally declared tribal area with the dominant population of Gaddi tribal community.

6 Several members of the Gaddi community have dual residences in two districts (i.e. Chamba and Kangra) of Himachal Pradesh. They migrate between these two locations depending upon the seasonal variations. Also, the practicing Gaddi pastoralists remain on the move throughout the year following a vertical transhumance.

7 Many pastoralists, especially in the mountainous regions, follow a vertical transhumance pattern in which their migratory routes remain fixed across the altitude following a seasonal rhythm.
